# Understanding the role of childhood abuse and neglect as a cause and consequence of substance abuse: the German CANSAS network

**DOI:** 10.1080/20008198.2017.1304114

**Published:** 2017-03-31

**Authors:** Ingo Schäfer, Silke Pawils, Martin Driessen, Martin Härter, Thomas Hillemacher, Michael Klein, Markus Muehlhan, Ulrike Ravens-Sieberer, Martin Schäfer, Norbert Scherbaum, Barbara Schneider, Rainer Thomasius, Klaus Wiedemann, Karl Wegscheider, Sven Barnow

**Affiliations:** ^a^ Department of Psychiatry and Psychotherapy, University Medical Center Hamburg-Eppendorf, Hamburg, Germany; ^b^ Center for Interdisciplinary Addiction Research, University of Hamburg, Hamburg, Germany; ^c^ Department of Medical Psychology, University Medical Center Hamburg-Eppendorf, Germany; ^d^ Department of Psychiatry and Psychotherapy, Ev. Hospital Bielefeld, Bethel, Bielefeld, Germany; ^e^ Department of Psychiatry, Socialpsychiatry and Psychotherapy, Hannover Medical School, Hannover, Germany; ^f^ Center of Excellence on Applied Addiction Research, Catholic University of Applied Sciences, Cologne, Germany; ^g^ Institute of Clinical Psychology and Psychotherapy, Department of Psychology, Technische Universität Dresden, Dresden, Germany; ^h^ Department of Psychology, Faculty of Human Science, Medical School Hamburg, Hamburg, Germany; ^i^ Department of Psychiatry, Hospital Essen-Mitte, Essen, Germany; ^j^ LVR-Hospital Essen, Department of Psychiatry and Psychotherapy, Faculty of Medicine, University of Duisburg-Essen, Essen, Germany; ^k^ Department of Addiction Medicine, LVR-Clinic Cologne, CologneGermany; ^l^ German Center for Addiction Research in Childhood and Adolescence, University Medical Center Hamburg-Eppendorf, Hamburg, Germany; ^m^ Department of Medical Biometry, University Medical Center Hamburg-Eppendorf, Germany; ^n^ Department of Clinical Psychology and Psychotherapy, Ruprecht-Karls-University Heidelberg, Heidelberg, Germany

**Keywords:** Trauma, maltreatment, neglect, posttraumatic stress disorder, addiction, substance use disorders

## Abstract

**Background**: Substance use disorders (SUD) belong to the most frequent behavioural consequences of childhood abuse and neglect (CAN). If parents are concerned, SUD are also an important risk factor for CAN. The relationship between CAN and SUD remains poorly understood. There is a need of adequate treatments for SUD patients suffering from the consequences of CAN, as well as for approaches to prevent CAN by parents with SUD.

**Objective**: To describe the aims and the structure of a German research network on relationships between CAN and SUD (CANSAS network).

**Method**: Descriptive overview of the aims, and the different project clusters of the network.

**Results**: The aims of the CANSAS network are: (1) to examine relationships between SUD and CAN; (2) to examine the effects of an evidence-based treatment for SUD patients with posttraumatic disorders; and (3) to provide SUD services with tools to diagnose CAN, and to assess the risk of maltreatment among parents with SUD. The aims of the network are addressed by six projects in three different project clusters (mediators and risk factors, evidence-based treatment, and improvement of services).

**Conclusions**: It is expected that the CANSAS network will advance the understanding of relationships between early adversity and substance use disorders. It will bring forward the discussion about promising treatments for SUD patients with experiences of CAN. Finally it will provide services with measures to identify patients with CAN, and with tools to break the trans-generational cycle of adversity.

## 1. Introduction

### 1.1. SUD as a consequence of CAN

Substance use disorders (SUD) belong to the most frequent behavioural consequences of childhood abuse and neglect (CAN). Of all patients seeking treatment for SUD, 16.3–60.9% report sexual abuse and 28.6–46.2% report physical abuse during their childhood (Simpson & Miller, 2002). Conversely, a lifetime diagnosis of SUD is found in 14–35% of adult survivors of CAN in community samples and in 30–35% of individuals who seek treatment for the consequences of CAN (Levitt & Cloitre, 2005; Mullen, Martin, Anderson, Romans, & Herbison, 1993).

### 1.2. Mediators and risk factors

Both psychological and biological factors seem to play a role for the development of SUD after experiences of CAN, but many aspects of this relationship remain poorly understood. Among the most important mediators are comorbid mental disorders, namely posttraumatic stress disorder (PTSD). Their important role for the development of SUD was initially suggested by proponents of the ‘self-medication hypothesis’ (Khantzian, 1985), and has been confirmed in many studies with different methodological approaches over the past two decades (Coffey, Stasiewicz, Hughes, & Brimo, 2006; Gielen, Krumeich, Tekelenburg, Nederkoorn, & Havermans, 2016; Kaysen, Stappenbeck, Rhew, & Simpson, 2014; Stewart, Conrod, Samoluk, Pihl, & Dongier, 2000). More recently, psychological consequences of CAN that could be important for different diagnostic entities have received increasing attention. This refers, for instance, to emotion regulation (ER) difficulties as a consequence of trauma (Ehring & Quack, 2010), which are also a potential predictor for SUD (Aldao, Nolen-Hoeksema, & Schweizer, 2010). Only few studies exist, however, on the interaction between ER deficits and subsequent craving and relapse in patients with SUD (Fox, Hong, & Sinha, 2008), and the impact of childhood trauma in this context (Banducci, Hoffman, Lejuez, & Koenen, 2014; Mandavia, Robinson, Bradley, Ressler, & Powers, 2016). Another promising perspective concerns alterations of the neuroendocrine responses to stress in victims of CAN (Tyrka, Burgers, Philip, Price, & Carpenter, 2013) that could also play an important role in the development of SUD (Enoch, 2011). Again, little empirical evidence exists regarding potential interactions between CAN and the neuroendocrine response to stress in patients with SUD (Moran-Santa Maria et al., 2010; Schäfer et al., 2010).

On a clinical level, PTSD is a frequent consequence of CAN in patients with SUD. Among patients treated for SUD, 26–52% have a lifetime diagnosis of PTSD and 15–41% currently meet criteria for the disorder (Schäfer & Najavits, 2007). In European studies, the rate of current PTSD is slightly lower, but still substantial (e.g. 15–36%; Driessen et al., 2008). However, PTSD is only one of the most obvious consequences of CAN and many SUD patients with a history of CAN suffer from more complex psychological consequences. They typically include pervasive disturbances in ER, a diminished and defeated sense of self, and difficulties in interpersonal relationships (Hien, Cohen, & Campbell, 2005). Such complex symptom presentations can dominate the clinical picture, or they can exist in addition to PTSD, which led the International Classification of Diseases (ICD) task force on stress-related disorders to propose a new diagnosis of ‘Complex PTSD’ for ICD-11 (Maercker et al., 2013).

### 1.3. Trauma-specific treatment of patients with SUD

SUD patients with a history of CAN present significant challenges to clinicians and the consequences of CAN have been related to poorer outcomes, lower treatment engagement and retention in this group (Bartholomew, Courtney, Rowan-Szal, & Simpson, 2005; Greenfield et al., 2002). With regard to specialized therapies for SUD patients with comorbidities related to CAN, preference is given to integrated treatments that conceptualize both disorders as related and plan treatment accordingly. While it is unclear if integrated treatments have a superior efficacy compared to one efficacious treatment alone (Torchalla, Nosen, Rostam, & Allen, 2012), the clinical needs of patients with SUD and PTSD often make such an integrated approach necessary. The most studied integrated treatment thus far is ‘Seeking Safety’ (SS; Najavits, 2002). This present-focused programme covers 25 topics that address the diverse consequences of CAN in four domains (cognitive, behavioural, interpersonal and case management) and has been shown to be effective in many SUD samples in the USA. The literature indicates, overall, positive outcomes on SUD and PTSD symptoms, as well as other consequences of CAN, in various populations (Najavits & Hien, 2013). A pilot study in Germany yielded positive results (Kaiser et al., 2015), suggesting that the programme could be a promising option also for European SUD settings, but no controlled studies on the efficacy of this treatment have been published in Europe so far.

It has been proposed that a trauma-informed organizational culture, that takes into account the role and impact of trauma in the patient’s lives, should be the basis for the implementation of trauma-specific services in health care settings (Brown, Harris, & Fallot, 2013). In current practice, however, this rarely seems to be realized. There is a wealth of evidence that health professionals often do not even ask about their clients’ experiences of CAN (Havig, 2008). The most important reason for this failure is an obvious lack of training for adequate inquiry and response to patients’ histories of abuse. For instance, in a large study among clinicians in community support centres, personal confidence and competence were positively related to the percentage of clients with whom trauma and PTSD had been discussed, documented, and addressed in treatment (Salyers, Evans, Bond, & Meyer, 2004). Other reasons seem to be a general underestimation of the prevalence of posttraumatic disorders in SUD patients and concerns to do harm when assessing trauma and PTSD (Gielen, Krumeich, Havermans, Smeets, & Jansen, 2014). Strategies to train practitioners regarding the consequences of trauma in their clients and regarding skills to assess CAN and other traumatic experiences therefor are a prerequisite for the dissemination of trauma-specific treatments in SUD settings.

### 1.4. SUD as a cause of CAN

Finally, SUD are not only a frequent consequence of CAN, but also belong to the most important risk factors for the perpetration of CAN. Studies of court registers indicate that about 40–60% of all significant CAN cases involve parents with substance use problems. A variety of factors seem to enhance the risk for CAN in families with SUD. Findings include dysfunctional internal and external boundaries, poor communication skills, high family conflict levels, and low levels of family competence (Vernig, 2011). One of the best-established hypotheses focuses on ‘disinhibition’ caused by active use of substances. Though under-studied so far, ER is emerging as another predictor for CAN among individuals with SUD. ER deficits can be linked to both addictive and aggressive behaviours (Simons & Carey, 2002) and they exceeded other diagnostic and demographic variables in predicting CAN among individuals with SUD (Hien, Cohen, Caldeira, Flom, & Wasserman, 2010). A prerequisite for efforts to prevent CAN by parents with SUD is effective screening of individuals at risk. The existing attempts to develop specific instruments for SUD populations, however, share some methodological shortcomings.

## 2. The CANSAS network

Some of the questions outlined above are currently addressed by the research network ‘CANSAS’,^1^ which is funded by the German Ministry for Education and Research. In a multi-disciplinary approach, the CANSAS network brings together experts in the fields of trauma treatment for patients with SUD, epidemiology and risk factor research, biological and psychological moderators, as well as health services research. The aims of the network are (1) to gain a better understanding of the relationships between the two important public health problems CAN and SUD; (2) to promote evidence-based treatments for survivors of CAN with SUD; and (3) to provide services with trainings to improve the assessment of CAN among clients with SUD and to assess risk factors for the perpetration of CAN in this population. The following sections give an overview of the six different projects of the CANSAS network.

### 2.1. Project cluster ‘mediators and risk factors’

The first cluster of the CANSAS network consists of projects with a focus on potential mediators between CAN and SUD in adult life. The project ‘Trauma, ER and substance-use disorder: does emotion dysregulation moderate the association between trauma and substance use/relapse?’ (PI Sven Barnow, Heidelberg) examines the mediating role of ER in the relationship between CAN and SUD. To this aim, *N *= 80 male and female healthy individuals with a history of CAN are compared with *N *= 160 SUD patients of both genders with such a history using different methodological approaches. This also includes ecological momentary assessment (EMA) to collect real-time data about subjects’ emotional status and ER strategies as they go about their lives within their normal environments. It is expected that traumatized SUD patients show ER deficits (e.g. more often use rumination, avoidance and less often reappraisal, show inflexibility) compared to traumatized healthy controls. Moreover, it is expected that ER deficits are related to characteristics of substance use as well as craving/relapse in currently abstinent traumatized SUD patients.

A second project (‘Relationship between CAN and neuroendocrine response to stress in individuals with SUD’; PI: Ingo Schäfer, Hamburg) examines relationships between CAN and neuroendocrine reactions to social stress in patients with alcohol dependence (AD). To this aim, *N *= 72 male and female AD patients (with and without childhood trauma) will be compared to *N *= 72 healthy controls (with and without childhood trauma) regarding their reactions to a social stress task (‘Trier Social Stress Test’; TSST). Other markers (e.g. hair cortisol levels) will be used to get a more holistic view of potential relationships between CAN and stress reactivity in AD patients and to identify their potential predictive value. It is expected that individuals with AD and CAN show decreased cortisol responses to the TSST as compared to AD individuals without CAN, the latter being comparable to healthy controls with CAN.

### 2.2. Project cluster ‘evidence-based treatment’

In the project ‘Cognitive-behavioural treatment for female patients with PTSD and SUD’ (PI Ingo Schäfer, Hamburg) *N *= 342 female patients with PTSD and SUD are included in a multi-centric study in Hamburg, Cologne, Essen, Bielefeld, and Hannover to compare the effects of SS (Najavits, 2002) to another standardized CBT intervention (relapse prevention training; RP), and treatment as usual (TAU). SS is suitable for patients of both genders, but this study will focus on female patients. While single-sex studies are clearly limited with regard to the generalizability of their findings (Kristman-Valente & Wells, 2013), this decision was made because of the higher prevalence of the comorbidity of SUD and PTSD in females (e.g. Driessen et al., 2008; Schäfer & Najavits, 2007), and the suggestion of some authors that trauma treatment should preferably be offered in gender-specific groups (e.g. Greenfield et al., 2007). Patients in the SS group receive 14 weekly sessions (90 min each) in groups of four to eight patients. The intervention is offered in addition to TAU. The same number and duration of sessions is offered to patients in the ‘RP + TAU’ group. Patients in the TAU group are offered individual SUD treatment on a weekly basis and may obtain any treatment they normally would (detoxification, rehabilitation, individual psychotherapy, self-help groups, etc.). While the primary outcomes are PTSD symptoms as measured by the PTSD Symptom Scale Interview (PSSI), the secondary outcomes include substance use and other domains of psychopathology. The primary hypothesis consists of three two-group comparisons: (1) Enhanced treatment (SS + TAU) is more effective with regard to reduction of PTSD symptoms at six-month follow-up than TAU alone; (2) the same is expected for relapse prevention training (RP) + TAU as compared to TAU alone; and (3) SS + TAU will be at least as effective as RP + TAU.

The second study of the cluster (‘Cognitive-behavioural treatment for adolescents with PTSD and SUD’; PI: Rainer Thomasius, Hamburg) examines the feasibility and efficacy of the adolescent version of SS in *N *= 74 adolescent girls diagnosed with PTSD related to CAN and substance use disorders. As in the first study of the cluster, the decision to focus on girls was made because of the higher prevalence of the comorbidity of SUD and PTSD in females, and the advantages related to offering trauma treatment in gender-specific groups. In this uncontrolled pilot study, it is expected that participants attending at least eight of 14 SS sessions (‘completers’) will show a significant reduction of posttraumatic symptoms at three-month follow-up as compared to baseline, as well as significant reductions of substance use.

### 2.3. Project cluster ‘improvement of services’

The project ‘Learning how to ask – evaluation of a training programme for practitioners in SUD services’ (PI: Martin Härter, Hamburg) aims at (1) adapting a standardized training programme for CAN inquiry and response (‘Learning how to ask’; Read, Hammersley, & Rudegeair, 2007) to the requirements of the German health care system; and (2) evaluating the effects of the programme on acceptance, knowledge about abuse in SUD clients, competence of abuse inquiry and the rate of abuse inquiry by practitioners in SUD services. The programme consists of one-day workshops and has been successfully used in New Zealand and in the UK (Read et al., 2007). The participants are provided with background information about forms and consequences of abuse, legal issues, and local possibilities for referral as well as guidelines for abuse inquiry and response. The effects of the training will be examined in a randomized-controlled trial among *N *= 120 practitioners of both gender in outpatient SUD counselling services in Hamburg. It is expected that the number of documented CAN inquiries is significantly higher in the six months post training in the intervention group as compared to the control group.

Another project of the cluster (‘Assessing the risk of CAN in parents with SUD: development of an evidence-based instrument’; PI: Ulrike Ravens-Sieberer, Hamburg) finally aims at the development and psychometric testing of a short risk inventory to identify the risk of CAN among parents with SUD. The instrument will be based on a meta-analysis of specific risk factors of families with parental SUD, a systematic assessment in 392 SUD counselling services representative for 1399 such services in Germany, as well as on qualitative interviews with different groups of SUD practitioners. The short risk inventory will be tested regarding acceptance and practicability in *N *= 40 SUD services in Hamburg.

## 3. Conclusion

Substance use disorders are not only one of the most significant consequences of violence and neglect in childhood, but, if parents are concerned, they are also one of the most important risk factors for maltreatment against children. Interdisciplinary research activities are necessary to identify the relationships between early adversity and substance use disorders, to provide effective treatments for patients with comorbid posttraumatic disorders and to inform preventive approaches. To our knowledge, the CANSAS network is the largest European research initiative in this field so far. It is expected that the findings of the network will lead to more sophisticated models regarding the role of early adversity for substance use disorders. First results confirmed the important role of emotions and their regulation and brought new insights into the details of this relationship (Wolff et al., 2016). The findings of CANSAS could thus provide a basis for early intervention approaches in children and adolescents with a history of CAN, and for specific ER trainings adapted to the needs of patients with SUD. The results of the studies on a treatment model for SUD patients with PTSD will bring forward the discussion about promising treatments for this group in European SUD settings. This includes the question of which patients can benefit from present-focused models like SS and which patients might need (additional) trauma-focused approaches. Finally, the findings on trainings of practitioners and screening tools will contribute to both, improving the assessment of CAN in patients with SUD and breaking the transgenerational cycles of adversity through a better assessment of CAN by parents with SUD. There are manifold relationships between CAN and SUD, and the network will only be able to make a limited contribution to this rapidly developing field of research. Nevertheless, it can be expected that the broad perspective of CANSAS and its interdisciplinary approach, that brings together experts in the fields of childhood abuse and neglect, substance use disorders, as well as health services research, will contribute to a better understanding of some relevant questions that might help to bring forward the idea of ‘trauma-informed care’ (Brown et al., 2013) in European addiction services.

## Note

1. 
**C**hildhood **A**buse and **N**eglect as a cause and consequence of **S**ubstance **A**buse – understanding risks and improving **S**ervices (**CANSAS**).
